# Dr. Adams, on Hydatids

**Published:** 1804-12-01

**Authors:** Joseph Adams

**Affiliations:** Village of Castle Douglas, near Dumfries


### To Dr. BRADLEY.

Dear Sir,

I Have heard it remarked, that your Miscellany grows less interesting than when first offered to the public. It certainly loses some of the advantages of novelty; and if any reader expects such a collection of original communications should contain no dull papers, we must be less surprised at his disappointment than his expectation. I am however persuaded, that the mistake arises, in great measure, from inattention. Men deeply engaged in business, are apt to take a hasty view of the table of contents, or even of the work itself; and if nothing particularly strikes their eye, the Number is laid down, like a newspaper in a coffee-room, with the common phrase, that *there is nothing new in it.*

To obviate as much as possible this indolence, to which we are all liable, would it not be advisable to be more attentive in your superscription to each article. I have been led to these reflexions in my present retired situation: for having access to few medical books, and much leisure, I have perused every article of your last Number with unusual industry. Nothing but such an event would have induced me to take notice of "Mr. Pulley's case of a dropsical enlargement of the abdomen." Whereas, had the paper been entitled, "*Case of hydatids in the cavity of the abdomen, in which the operation of tapping was performed; with an account of the appearances on dissection;*" few of your medical readers would have passed it over; and, to me, few subjects would have been more interesting. Having selected this paper, I cannot dismiss it without a few remarks, which I doubt not the writer will peruse with the same candour as is conspicuous in every part of his communication.

Hydatids, from the cause he describes, are by no means uncommon, not only in the abdomen, but in other parts of the body. If I am incorrect in my quotations, you will recollect they can only be from memory. A case similar to Mr. Pulley's is given by Dr. Lettsom in one of the volumes of Memoirs of the Medical Society; another occurs in the Edinburgh Medical Essays, and, I believe, in the Medical Transactions. I have given three cases in which hydatids were traced from violence in different parts of the body; one on the authority of Dr. Stokes, another witnessed by Mr. Abernethy and myself at St.

Bartho-

Bartholomew's, and a third in the abdomen from a blow. But what I wish particularly to take notice of, is the remark with which the paper concludes. "Whatever," says this equally modest and ingenious writer, "may be the prevailing opinion concerning the formation of hydatids, it would appear, in this instance, that they took their origin from the accident. Inflammation of the peritoneum, doubtless, was the consequence of the kick, and probably an effusion of coagulable lymph, the birth of cystic dropsy. The largest sac was connected with the part that received the inflicted blow; most probably, it was the first formed, and from its rapid increase, possibly it assumed an inflammatory state, and by an effusion of coagulable lymph from its inner surface, might give rise to the existence of those minor hydatids, which were found floating within its cavity. This sac possessed much firmness and density, and certainly was highly vascular."

Nothing can be more reasonable than the conclusion, that the kick and consequent inflammation were the causes of the formation of hydatids. But how can we assume from such premises, that inflammation and the effusion of coagulable lymph should produce a *multiplication* of uniform substances, filled with a transparent fluid that is not coagulable. If this multiplication were the effect of fresh inflammation and effusion, should we not have found the usual symptoms of inflammation occasionally occur? which does not appear by the history of the case. If we admit, as seems almost demonstrated by Dr. J. Hunter, that the hydatid is an animal possessing a life independent of the body in which it grows, excepting by the nourishment it absorbs, we shall I conceive more readily comprehend their multiplication by the analogy we find in other less complicated animals. If any part of the body is rendered useless by a cause which does not deprive it of life, nothing seems more consistent with the economy of nature than that such a part should become the nidus of a race of animals, that can only exist in living animal matter. Hence, if lymph is thrown out, and instead of being reabsorbed, retains its life, and assumes vessels for its support, the same consequences may be expected.

Your readers, and particularly Mr. Pulley, will consider these remarks in no other light than as hints against assigning causes which, without further reasoning, do not appear equal to the effect. If Dr. Hunter's theory of hydatids is thought inconclusive, it seems at least reasonable to to offer some objections when a new one is started. I trust, however, I shall not be thought to undervalue Mr. Pulley's communication, when my only wish is that he should dilate with more confidence on his own opinions.

I shall conclude this paper with some account of the result of my inquiries concerning the cow-pox inoculation during a journey through all the North-West counties, from Bristol to New Galoway. At every town I have taken pains to be introduced to the different practitioners, in order to gain information of anomalous cases by which to ascertain every law in morbid poisons. Sanguine as I was in favour of the practice, I could not help being astonished at the success with which it has been uniformly attended. The deviations from the established laws of the disease have been so few as to afford me no new facts.

The mention of the cow-pox must be my apology if I do not yet conclude. Dr. Jenner has a valuable collection of facts relative to the hydatids and other diseases in brutes. It is much to be wished that he would give them to the world, and at least ascertain the precise characters of the more common maladies to which brutes are liable. Even the grease in the horse is not yet clearly characterized; and the want of precision in this respect has given rise to much misconception and controversy. Let us hope the ingenious writer will not be discouraged from again appearing before the public by those severities which every reformer should prepare himself to receive.

I remain, &c.

JOSEPH ADAMS.

Village of Castle Douglas, near Dumfries, Nov. 1, 1801.

